# Targeted, LCMS-based Metabolomics for Quantitative Measurement of NAD^+^ Metabolites

**DOI:** 10.5936/csbj.201301012

**Published:** 2013-05-27

**Authors:** Samuel AJ Trammell, Charles Brenner

**Affiliations:** a Department of Biochemistry; b Interdisciplinary Graduate Program in Genetics Carver College of Medicine, University of Iowa, Iowa City, IA 52242, USA

## Abstract

Nicotinamide adenine dinucleotide (NAD^+^) is a coenzyme for hydride transfer reactions and a substrate for sirtuins and other NAD^+^-consuming enzymes. The abundance of NAD ^+^, NAD^+^ biosynthetic intermediates, and related nucleotides reflects the metabolic state of cells and tissues. High performance liquid chromatography (HPLC) followed by ultraviolet-visible (UV-Vis) spectroscopic analysis of NAD^+^ metabolites does not offer the specificity and sensitivity necessary for robust quantification of complex samples. Thus, we developed a targeted, quantitative assay of the NAD^+^ metabolome with the use of HPLC coupled to mass spectrometry. Here we discuss NAD^+^ metabolism as well as the technical challenges required for reliable quantification of the NAD^+^ metabolites. The new method incorporates new separations and improves upon a previously published method that suffered from the problem of ionization suppression for particular compounds.

## Introduction

The essentiality of NAD^+^-dependent processes in fuel utilization, gene regulation, DNA repair, protein modification, and cell signaling events makes the analysis of NAD^+^ metabolites central to an understanding of what a tissue is doing. NAD^+^ is the key hydride transfer coenzyme for a wide variety of oxidoreductases and is also the consumed substrate of sirtuins, poly adenosine diphosphate ribose (ADPr) polymerase, mono ADPr transferases, and cyclic ADPr synthases [1, 2]. Measurement of NAD^+^ and related metabolites including several nucleosides and nucleotides (hereafter, the NAD^+^ metabolome) serves as a powerful indicator of the ability of a cell or tissue to perform processes such as glycolysis, gluconeogenesis, fatty acid oxidation, reactive oxygen species detoxification, among others. Moreover, the state of the NAD^+^ metabolome can serve as an indication of nutrition, health and disease.

Because NAD^+^ and related metabolites vary in cellular concentration from ∼1 µM to ∼1 mM, the analytical procedure must be robust, reproducible, and rapid. Liquid chromatography (LC)-based assays afford the ability to measure multiple metabolites in a timely fashion with the duration of each run ranging from 10 minutes to an hour. However, quantification through HPLC-UV-Vis methods is severely compromised based on the complexity of samples. In complex mixtures, a single peak may contain the metabolite of interest in addition to many other metabolites of identical retention time. In addition, peak shapes are rarely unaffected by complexity. Some investigators use a UV-vis signal at a retention time as the primary means for identification of a metabolite of interest—collected fractions are then subjected to mass spectrometry to confirm (nonquantitatively) the presence of the metabolite. This process leaves a great deal of data in the dark. Because every NAD^+^ metabolite can be converted to one or more other metabolites, snapshots of the levels of NAD^+^, nicotinamide (Nam) or any other NAD^+^ metabolite without assessment of the NAD^+^ metabolome on a common scale has the potential to be misleading.

Because of its specificity and sensitivity, LC coupled to mass spectrometry (LC-MS) is a leading analytical method in the measurement of small molecules in complex samples. As with HPLC-UV-vis methods, LC serves to separate compounds of interest and must be optimized in the same way as any HPLC method. Because all LC-MS data contain at least two dimensions of data (retention time plus the mass:charge ratio, termed *m/z*), LC-MS increases specificity with respect to LC-UV-vis methods that report complex absorbance spectra as a function of retention time or matrix-assisted laser desorption ionization (MALDI)-based methods that report complex *m/z* data without retention times. Multidimensional MS, *i.e*., LC-MS^n^, provides further information because a particular analyte breaks down to component ions at a particular ionization energy. An ideal LC-MS method identifies an optimal extraction and separation method for all molecules of interest, detects the compounds in either negative or positive ion mode MS, and has sufficient LC separation to subject each molecule of interest to MS^n^ analysis. The method is then a series of selective reaction monitoring (SRM) protocols in which analytes are identified and quantified by MS as they come off the LC.

Whereas metabolomic discovery projects require high mass resolution instruments, targeted quantitative LC-MS assays can make use of lower resolution tandem mass spectrometers such as triple quadrupoles (QQQ). Here, the multidimensional data (retention time, *m/z*, and MS^2^ transitions) are used to distinguish closely related metabolites, such as NAD^+^ from NADH. Limits of quantification in optimized targeted, quantitative LC-MS assays are in the femtomole range.

Though mass spectrometers offer great analytical power for measuring the NAD^+^ metabolome, they also present technical challenges not encountered in other analytical techniques. These challenges include development of optimal mass spectrometry conditions, proper separation of metabolites, and the best choice of internal standards. Here we discuss NAD^+^ metabolism and describe an optimized LC-MS^2^ assay of the NAD^+^ metabolome.

## NAD^+^ Transactions

**Figure 1:**
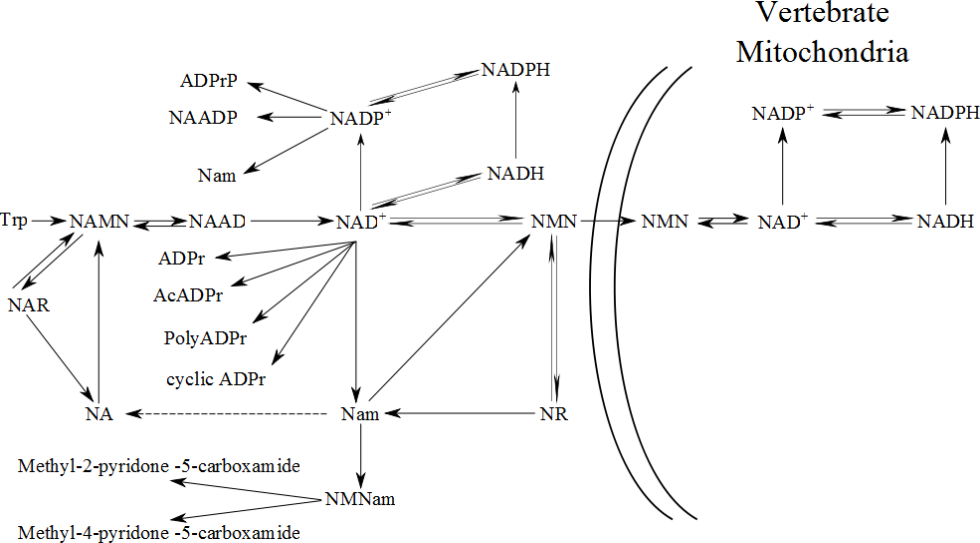
**NAD^+^ Biosynthesis in yeast and vertebrates**. Intracellular NAD^+^ is derived from either *de novo* synthesis from tryptophan or from salvage of NA, Nam, or NR. In yeast, Nam is converted to NA by nicotinamidase Pnc1p (dotted line). In yeast and vertebrates, NA is phosphoribosylated to NAMN, an intermediate in *de novo* synthesis, and converted to NAD^+^ by way of NAAD in a step catalyzed by glutamine-dependent NAD^+^ synthetase [12]. In vertebrates, Nam conversion to NMN is catalyzed by Nampt [16]. The other source of NMN in yeast and vertebrates is phosphorylation of NR by NR kinases. NR and NAR can be split to the corresponding pyridine bases. NAR phosphorylation yields NAMN. NMN is converted to NAD^+^ by NMN adenylyltransferase activity, which is reversible. As shown, in vertebrates, NMN must be imported into mitochondria for conversion to NAD^+^. Enzymatic NAD^+^ and NADP^+^ consumption releases the Nam moiety and produces ADPr products. Finally, Nam and NA can be converted to non-salvageable products.

In fungi and vertebrates, NAD^+^ concentration is maintained by either *de novo* synthesis from tryptophan [4] or through salvage of nicotinic acid (NA) [5], nicotinamide (Nam) [6], and the recently identified NAD^+^ precursor vitamin, nicotinamide riboside (NR) [7] (Figure 1). Some organisms, such as *Candida glabrata*, lack *de novo* synthesis [8]. Many vertebrate cell types turn this pathway off [2]. *De novo* synthesis proceeds from tryptophan in six steps to produce nicotinic acid mononucleotide (NAMN) and in two additional steps to produce NAD^+^. When NAD^+^ is the substrate of an enzyme such as glyceraldehyde phosphate dehydrogenase (GAPDH), fuel oxidation reactions will reduce NAD^+^ to NADH. In the case of GAPDH, the reaction is reversible, such that NADH is reoxidized to NAD^+^ in the gluconeogenic direction. NAD^+^ and NADH can be phosphorylated to NADP^+^ and NADPH. NADP^+^ is required for the pentose phosphate pathway (PPP), which produces NADPH. NADPH is required for detoxification of reactive oxygen species and reductive biosynthesis of lipids and steroids. Just as glucose-6-phosphate oxidation by the PPP produces NADPH, glutathione reactivation and reductive biosynthesis reoxidizes NADPH to NADP^+^.

Beyond serving as a coenzyme in hydride reactions, NAD^+^ is a consumed substrate for enzymes such as sirtuins, PARPs, and other ADPr transfer enzymes [1, 2, 9, 10]. Though CD38 has an activity on NADP^+^, at least *in vitro* [11], the typical activity of an NAD^+^-consuming enzyme involves NAD^+^ as the substrate, and products that include Nam and an NAD^+^-derived ADPr moiety. Thus, to sustain intracellular NAD^+^ levels, actions of NAD^+^-consuming enzymes must be accompanied by Nam salvage [1, 2]. Nam salvage differs between fungi and vertebrates. In fungi, Nam is hydrolyzed by the *PNC1*-encoded nicotinamidase to NA [6]. NA is then converted by the first enzyme of the Preiss-Handler pathway, the *NPT1*-encoded NA phosphoribosyltransferase, to form NAMN. The second and third steps of Preiss-Handler salvage correspond to the final two steps of *de novo* synthesis, whose last step is glutamine-dependent NAD^+^ synthetase [12]. In vertebrates, Nam produced as a product of NAD^+^-consuming enzymes cannot be salvaged as NA intracellularly. However, if Nam goes through the gut, bacterial nicotinamidases produce NA [13], which circulates and is used *via* Preiss-Handler salvage.

Intracellular Nam salvage in vertebrates depends on a Nam phosphoribosyltranferase, which entered the scientific literature with the names pre-B cell colony enhancing factor (PBEF) [14] and Visfatin [15]. Now termed Nampt, this protein is widely expressed as an intracellular enzyme and also circulates as an active extracellular molecule [16, 17]. First predicted to be part of a partially extracellular NAD^+^ biosynthetic cycle [1] along with CD73, a homolog of bacterial NMN 5’-nucleotidase, extracellular Nampt clearly has enzymatic activity [17]. However, extracellular NMN remains controversial in part due to deficiencies in NAD^+^ metabolite quantification. As a phosphoribosyltransferase, Nampt activity depends on phosphoribosyl pyrophosphate (PRPP), an extracellular source of which has not been demonstrated [18]. By an HPLC-UV method, which may have been distorted by co-eluting analytes, the abundance of extracellular NMN was reported to be 80 µM [17]. However, using LC- MS^n^, it was reported that PRPP and NMN are virtually absent and, moreover, are unstable in mouse plasma [18]. It stands to reason that extracellular Nampt may have activity in local environments and developmental/nutritional conditions in which the substrates, Nam and PRPP, and the ATP activator are at substantial levels. Systemic NMN at 80 µM appears to be implausible, however.

Nam and NA can also be methylated, which would be predicted to block salvage. In plants, NA *N*-methyltransferase produces a compound known as trigonelline by transfer of the methyl group from *S*-adenosyl-methionine [19, 20]. The corresponding Nam *N*-methyltransferase (NNMT) has been well characterized in vertebrates [21]. Increased NNMT expression has been observed in Parkinson's Disease [22] with a potential role in disease etiology [23, 24]. NNMT is also increased in malignancy [25] and plays an apparent role in cell migration [26]. Despite the reported roles in disease, *N*-methyl Nam (NMNam) is a natural metabolite in healthy individuals with reported antithrombotic [27] and vasorelaxant [28] activities that is increased in plasma and urine after endurance exercise [29]. NMNam is ultimately converted to *N1*-Methyl-2-pyridone-5-carboxamide and *N1*-Methyl-4-pyridone-5-carboxamide.

Though the primary breakdown product of NAD^+^ is Nam and the complete bacterially digested product is NA, nicotinamide riboside (NR) is an additional salvageable precursor that exists intracellularly and in milk [3, 7, 30]. The unique NR salvage pathway is *via* nicotinamide riboside kinases [7]. In addition, NR can be split into a Nam moiety and resynthesized to NAD^+^ *via* Nam salvage enzymes [31]. Nicotinic acid riboside (NAR) is an alternate substrate of nicotinamide riboside kinases [32] and purine nucleoside phosphorylase [13] that has been shown to be an intracellular NAD^+^ precursor [30] but has not been reported to circulate.

Whereas NA is the salvageable precursor of NAD^+^ that has been exposed to the most digestive enzymes and Nam is the salvageable precursor that is produced by every cell with NAD^+^-consuming enzymes, the main source of dietary NR is probably partial digestion of NAD^+^. Depending on one's nutrition and potentially one's microbiome, the three vitamin precursors of NAD^+^ (NA, Nam and NR) and trp should be in circulation [2]. The existence of extracellular enzymes with the potential to produce and consume NMN, and which consume NAD^+^, suggests the circulation of pyridine nucleotides [1]. Moreover, NMN supplementation of mice on high fat diet (HFD) increases insulin sensitivity, glucose tolerance, and intracellular NAD^+^ compared to non-treated mice on the same diet [33]. Though extracellular NMN was interpreted to function *via* direct incorporation of the nucleotide into cells [33], careful examination indicates that extracellular NA, Nam, and NR increase intracellular NAD^+^ in yeast and vertebrate cells, whereas NMN requires dephosphorylation to NR [34]. Consistent with the prediction that the ectoenzyme CD73 has NMN 5’-nucleotidase activity [1], CD73 has the requisite biochemical activity to catalyze NMN dephosphorylation [35]. In the yeast system, NR extends replicative longevity in a manner that depends on conversion to NAD^+^ [31]. In mice on high fat diet, NR improves glucose control and insulin sensitivity, while moderating the observed increase in adiposity [36].

In addition to the major difference in Nam salvage between vertebrate and yeast systems, there is a mitochondrial compartmentalization problem in vertebrates. In yeast, transporters Ndt1 and Ndt2 carry NAD^+^ across the mitochondrial inner membrane [37] and the only mitochondrial NAD^+^ biosynthetic enzyme is NADH kinase, Pos5 [38]. However, in vertebrate cells, the nucleocytoplasm and the mitochondrial matrix constitute distinct pools of NAD^+^, NADH, NADP^+^ and NADPH owing to impermeability of the mitochondrial inner membrane to these compounds. Though systems such as the malate-aspartate shuttle and nicotinamide nucleotide transhydrogenase transfer reducing equivalents across mitochondrial membranes, vertebrate mitochondria require a system to import an NAD^+^ precursor into the matrix for conversion to NAD^+^. On the basis of localization of NAD^+^ biosynthetic enzymes, that precursor is NMN [34]. Nmnat3, which converts NMN to NAD^+^, is localized to the mitochondrial matrix. Nmnat3 is one of three vertebrate NAMN/NMN adenylyltranferases—the other two are localized in the nucleus and on the cytosolic face of Golgi. Though one could argue that the ability of Nmnat3 to convert NAMN to NAAD suggests that NAMN or NMN could be the mitochondrial NAD^+^ precursor, the NAAD product of the NAMN reaction requires glutamine-dependent NAD^+^ synthetase for conversion to NAD^+^. Glutamine-dependent NAD^+^ synthetase is not mitochondrially localized [34].

As shown in Figure 1, the implication of NMN as the limiting precursor for vertebrate mitochondrial NAD^+^ biosynthesis is profound. *De novo* synthesis and NA-dependent Preiss-Handler synthesis can only supply mitochondria with NAD^+^ by nucleocytosolic conversion to NAD^+^ followed by the pyrophosphate-dependent conversion of NAD^+^ to NMN in a back reaction of Nmnat first demonstrated by Arthur Kornberg in 1948 [39] or by conversion of NAD^+^ to Nam and subsequent conversion of Nam to NMN. In contrast, Nam and NR can be converted directly to NMN by Nampt and NR kinases, respectively.

In mitochondria that are burning fuel, the redox reactions are largely directional because fuel oxidation converts NAD^+^ to NADH and complex I of the electron transfer chain reoxidizes NADH to NAD^+^. Three vertebrate sirtuins, Sirt3-5, reside in mitochondria, where they consume NAD^+^ in reactions that either modify proteins or relieve protein modifications [40]. For the mitochondrial sirtuins to work and avoid robbing redox enzymes of NAD^+^, NMN must be imported from the cytosol.

Completing the major NAD^+^ transactions, yeast possess two cytosolic NAD^+^/NADH kinases and the mitochondrial NADH kinase, Pos5 [38]. Vertebrate cytosolic NAD^+^/NADH kinase is related to the yeast enzymes [41], whereas the vertebrate mitochondrial NAD^+^/NADH kinase was recently identified as a homolog of *A. thaliana* Nadk3, which can use ATP or polyphosphate as the phosphate donor [42].

## Quantitative NAD^+^ Metabolomics

The NAD^+^ metabolome, as defined here, includes dinucleotides, nucleotides, nucleosides, nucleobases and related compounds (Table 1). The masses of many of the analytes differ by a single Dalton, necessitating optimal separation and careful MS. The current method is an improvement over methods, which measured only select metabolites [17, 43], and more recent methods, which embraced a more complete set of metabolites, but which lacked resolution of several compounds [3, 44]. Here we review optimization of all parameters and a solution to the ionization suppression problem that plagued previous methods.

**Table 1:** Alkaline separation gradient.

Time (min)	Solvent B (%)	Column Volumes
0	5	-
1.8	5	1.8
14	54	12.4
14.1	90	-
17.1	90	3
17.2	5	-
32.2	5	15.3

### Optimized extraction

Methods that do not inactivate enzymatic activities upon cell lysis [44] are clearly flawed and, based upon the amount of time of sample autolysis, cellular NAD^+^ can be degraded to ∼1% of expected values (∼10 µM) with elevation of apparent NR concentration to ∼100 times expected values (1 mM) [45]. The preferred method of extraction is to use boiled, buffered ethanol [46], which is well validated for NAD^+^ metabolites [3, 30].

For yeast samples, an ideal cell number is 2 to 4.5 x 10^7^, the midrange of which can be obtained by harvesting 25 ml of cells at an OD_600__nm_ of 0.7. For mammalian cell culture, we typically use 4 to 20 x 10^6^ cells, depending upon the cell type. Yeast cell pellets are extracted directly. Mammalian cell pellets are washed once in ice-cold potassium buffered saline. Cells are resuspended in 300 µL of a 75% ethanol/25% 10 mM HEPES, pH 7.1 v/v (buffered ethanol) solution, preheated to 80 °C. Samples are shaken at 1000 rpm in an 80 °C block for three minutes. Soluble metabolites are separated from particulate by refrigerated microcentrifugation (10 min, 16k*g*). Though the ethanol-soluble extract contains all the metabolites of interest, the weight of the particulate can be used to determine the optimized resuspension volume for dried metabolites. Thus, both the particulate and soluble metabolites are dried by speed vacuum at 40 °C.

Empirically, we determined that 3.6 mg of yeast or mammalian cell-derived particulate corresponds to a metabolite pellet, which can be resuspended in a 100 µl volume and produce the desired absorbance and LC-MS signals. Thus, the dry weight of each pellet is recorded, divided by 3.6 mg, and multiplied by 100 µl to obtain the initial resuspension volume. Extracts are resuspended in 1% (v/v) acetic acid adjusted to pH 9 with ammonium hydroxide (ammonium acetate buffer). These conditions were chosen to preserve NADH and NADPH prior to analysis [47].

Resuspended metabolites (2 µl) are checked in a Nanodrop (ThermoFisher) to determine the OD_260__nm_, which is typically greater than or equal to 14. The remaining volume is diluted to an OD_260__nm_ of 14 in ammonium acetate buffer to obtain the final resuspension volume. For LC-MS, this material is diluted two-fold into two different metabolite standards and 2.5 µl of the resulting material is injected and analyzed. Because these dilutions convert the total intracellular volume into a known volume of which an effective volume of 1.25 µl is analyzed, it is straightforward to calculate the intracellular volume of cells under analysis.

The calculation of intracellular volume is as follows. For yeast cells, the intracellular volume of a single cell is taken as 70 fl [48]. Thus, the calculated intracellular volume is obtained as 70 fl times the cell number. For mammalian cells, we use 2.5 pl as volume of a HeLa cell [49] and calculate the total extracted intracellular volume in the same way as for yeast cells. For example, an extraction of 3 x 10^7^ yeast cells has a calculated intracellular volume of 2.1 µl. If this sample were resuspended into 100 µl and require no further adjustment after checking on the Nanodrop, the 1.25 µl of cell extract in a 2.5 µl injection would represent 1.25% of 2.1 µl = 26 nl. Because the internal standards permit metabolites to be quantified on a mol scale, intracellular metabolite concentrations are determined, in this example, as mol of metabolite divided by 2.6 x 10^-8^ l.

### Optimized internal standards

Ionization suppression is the tendency for sample components to dampen the ionization and detectability of particular analytes. Thus, one cannot reliably depend on the peak height or area of a metabolite in a standard curve of purified metabolites to be on the same scale as its peak size in a complex mixture. In the most advanced previous quantification method for NAD^+^ metabolites, ionization suppression was a problem for NAD^+^, inosine and NA [3].

Two internal standard sets are employed. One set is used to quantify 16 metabolites (all analytes except NR, Nam, and NA) and is used in an alkaline separation. The other set is used to quantify NR, Nam, and NA in an acidic separation.

For analytes in the alkaline separation, an extract of Fleischmann's yeast metabolites is prepared from cells grown in 99% uniformly labeled ^13^C glucose (Icon Isotopes, Summit, New Jersey). In the course of this culture, the PPP converts ^13^C glucose to ^13^C ribose-5-phosphate, such that all of the cells’ nucleic acids, mononucleotides and dinucleotides incorporate ^13^C. Mononucleotides incorporate 5 additional Da from one ribosyl moeity, whereas dinucleotides incorporate 10 additional Da from two ribosyl moeities. Complete incorporation is achieved by growing two serial starter cultures in ^13^C glucose synthetic dextrose complete media, followed by inoculation of a 250 ml volume of ^13^C glucose media at a starting OD_600_ of 0.2 and growth to OD_600_ of 0.8. Cells in 50 ml aliquots are then pelleted and stored at -80 °C prior to extraction with 300 µl buffered ethanol solution. Metabolites are resuspended in 100 µl of the ammonium acetate buffer and have a typical OD_260__nM_ of 100. For LC-MS, Fleischmann's extract is diluted 1:40 into ammonium acetate buffer. This diluted Fleischmann's extract is further diluted 1:1 with experimental samples. In the LC-MS analysis, 2.5 µl of the fully diluted material is injected. This material represents 1.25 µl of the experimental extract and an appropriate amount of Fleischmann's extract for metabolite quantification.

Because the vitamins Nam and NA do not contain a carbohydrate group, they are not labeled by heavy labeled glucose and require the second set of internal standards for accurate quantification. To each sample or standard solution, heavy labeled Nam and NR are added such that the final concentration is 1.5 µM. ^18^O labeled Nam is prepared by tetramethylguanidine- catalyzed hydrolysis with 3-cyano-pyridine and H_2_^18^O, as described [50]. Though heavy NR is present in the Fleischmann's yeast extract standard, NR is best separated and quantified in an acidic separation with Nam and NA. Heavy labeled NR is made as described [51].

We prepare stock solutions (typically at 10 mM) of each metabolite to be quantified and then prepare a set of standard solutions containing the whole set of metabolites to be quantified at a range of concentrations (0, 0.1, 0.2, 0.6, 2, 6, 20, 60, and 200 µM). For the alkaline separation, these standards are mixed 1:1 with the 1:40 dilution of Fleischmann's extract so that each quantifiable metabolite in the extract can be set to a pmol amount. For example, if a particular metabolite in the Fleischmann's sample were to interpolate precisely between the 2 µM and 6 µM standards (1 µM and 3 µM) in the 2.5 µl injected volume, we would calculate there to be 5 pmol of that metabolite in the standard amount of Fleischmann's extract that will be used for all subsequent samples. This determination is made in technical replicates. Importantly, we cannot expect the 5 pmol peak area of that metabolite to remain constant when Fleischmann's extract is mixed with experimental yeast extracts because peak shapes are often distorted, and ionization is suppressed due to sample complexity. However, because the ^13^C metabolites in Fleischmann's extract will have the same degree of ionization suppression as the ^12^C metabolites in the experimental extract, the peak area ratios of ^12^C to ^13^C metabolites and the known mol amounts of the Fleischmann's metabolites allow calculation of the amounts of the experimental metabolites. Molar calculation of the Fleischmann's metabolites are not performed. Mol amounts of metabolites in the experimental samples are converted to molar using the calculation of intracellular volume described above. Because the IMP peak in Fleischmann's extract is quite small, we use relative peak areas of IMP and NMN in the standard solutions to derive a correction factor that allows IMP in experimental samples to be quantified against the Fleischmann's NMN peak. For each metabolite, the peak area used for quantification is that of the MS^2^ transition.

In theory, inclusion of 1.5 µM ^18^O labeled Nam and ^18^O labeled NR should be sufficient to quantify nonlabeled Nam and NR in the experimental extracts. In practice, because label incorporation may vary and there is greater accuracy in preparation of 10 mM standards than 1.5 µM radiolabeled standards, we quantify the ^18^O peaks of Nam and NR against a standard curve of NA, Nam and NR. These analyses result in a mol amount of heavy Nam and NR determined from the light standards. Because the same amount of heavy Nam and NR will be in all experimental samples, relative peak areas allow conversion of experimental Nam and NR peaks to mol and molar. The peak area ratio between heavy Nam and NA in the standard solutions is used to derive a correction factor that allows the heavy Nam peak to calculate the amount of NA in experimental samples.

### Optimized liquid chromatography

Earlier, we described an assay of the NAD^+^ metabolome based on hydrophilic interaction liquid chromatography [3]. Encouraged by its track record for separation of nucleosides and nucleotides [52–54], we have since developed an improved separation with the porous graphitic carbon reversed phase material, Hypercarb (Thermo). Resolution of all compounds is done with two different mobile phases on two Hypercarb columns, each used solely for one separation.

In the alkaline separation, solvent A is 7.5 mM ammonium acetate with 0.05% (v/v) ammonium hydroxide and solvent B is 0.05% (v/v) ammonium hydroxide in acetonitrile. The optimized gradient is described in Table 1 with a flow rate of 0.08 ml/min and a column temperature of 60 °C. As shown in Table 1, the complete run takes 32.2 min on a 1 mm x 100 mm Hypercarb column and must be equilibrated for 20 min prior to first injection.

**Figure 2:**
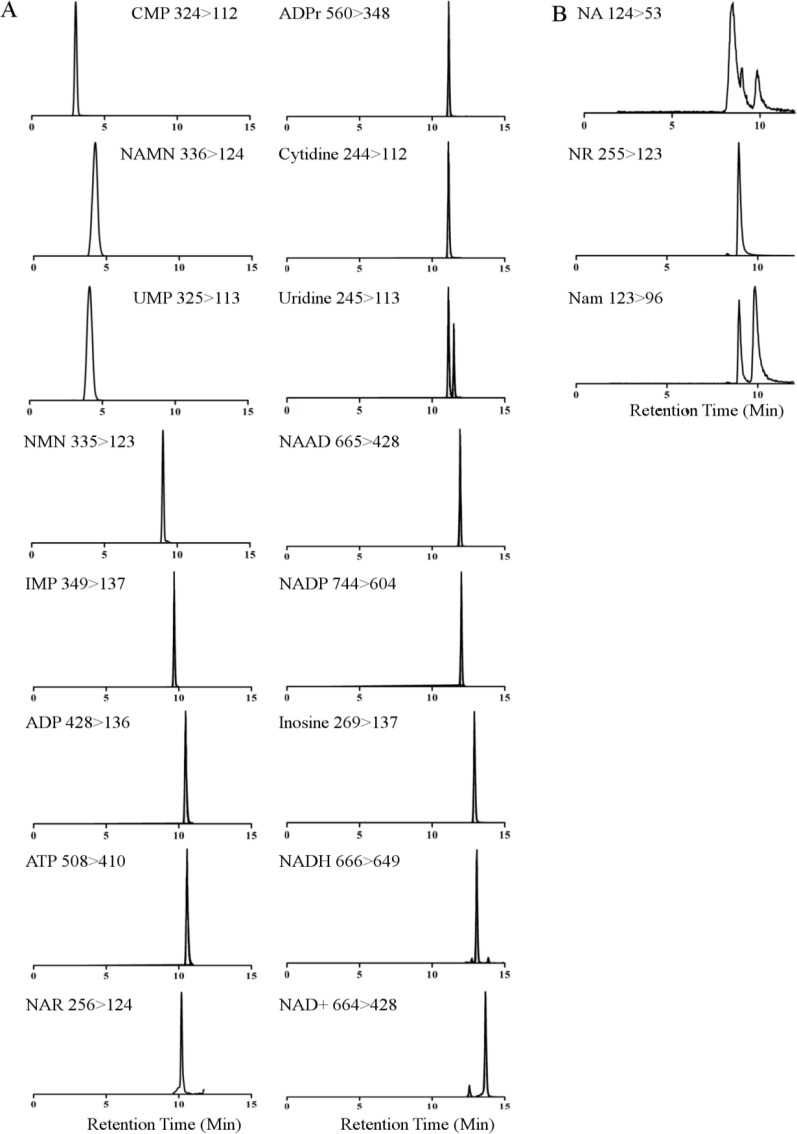
**Chromatograms of all 19 metabolites generated from injection of complex standard solutions using MRM LC-MS**. All compounds were detected in positive ion mode in alkaline (A) and acidic (B) separations. Multiple peaks observed in Uridine and NA illustrate cross talk from other metabolites in mixture. In the case of Uridine, the early eluting peak is the result of the ^13^C peak of Cytidine. NA later eluting peaks are the result of ^13^C peaks from Nam produced either from the Nam standard or on-source fragmentation of NR to Nam.

In the acid separation, solvent A is 10 mM ammonium acetate with 0.1% formic acid and solvent B is 0.1% formic acid in acetonitrile. The optimized gradient is described in Table 2 with a flow rate of 0.2 mL/min and a column temperature of 60 °C. As shown in Table 2, the complete run takes 23.4 min on a 2.1 mm x 100 mm Hypercarb column. Extracted ion currents for each resolved metabolite are provided in Figure 2.

**Table 2:** Acidic separation gradient.

Time (min)	Solvent B (%)	Column Volumes
0	5	-
1.8	5	1
11.2	35.9	5.4
11.3	90	-
13.3	90	1.2
13.4	5	-
23.4	5	5.8

### Mass Spectrometry Optimization

The power of triple quadrupole mass spectrometers is the ability to perform multiple SRM protocols in a single run, *i.e*. multiple reaction monitoring (MRM). Modern QQQs, such as the Waters Acquity TQD employed herein, are equipped with automatic optimization software to detect transitions and optimize ionization. The software can be the best friend and greatest enemy in method development. Since many of the metabolites are of similar structure and mass, specific diagnostic fragments must be identified and optimized. The carboxylic acid versus carboxamide metabolites and the oxidized versus reduced metabolites differ by only one Dalton. The ^13^C peaks produced from metabolites such as NAD^+^ and NADP^+^ would produce crosstalk with NADH and NADPH, respectively (Figure 2A). However, all four compounds produce diagnostic fragments, allowing for specific quantification. Current automatic optimization software identifies fragments that are most easily produced and not necessarily those that are diagnostic for the metabolite in the context of structural similarities in the NAD^+^ metabolome. Online searchable libraries such as Metlin (http://metlin.scripps.edu/) and Massbank (http://www.massbank.jp/?lang=en) provide MS/MS spectra for many metabolites with identified fragment structures [55, 56]. Specific fragments for NADH and NADPH not identified by the automatic software were chosen based on these searches. The transitions were optimized manually. Transitions and optimized conditions are detailed in Table 1.

The cone voltage must be optimized when measuring NAD^+^ especially for NR, NAR, NAMN, and NMN to reduce on-source fragmentation. NR and NMN readily produce Nam signal, whereas NAR and NAMN produce NA signal (Figure 2B, Nam extracted ion current). Optimization of cone voltage decreases but does not completely remove crosstalk. This unavoidable crosstalk greatly illustrates the need for robust LC separation.

After development, overall robustness was determined based on the capacity factors (*k’*), quantitative range, linear goodness of fit (R^2^), replicative standard deviation (RSD) of the method, and RSD of the system. Capacity factors were above 2 for all analytes with the exception of CMP (*k’* = 1) (Table 1). Standard curves were linear from 0.125 picomoles to 250 picomoles with R^2^ values falling above 0.99 for all but NAR (0.948), inosine (0.974), NR (0.981) and NADH (0.988). RSD of the method was measured with six separate standard solutions at 10 µM concentrations. RSD of the system was measured with four injections of the same standard solution. Method RSDs were below 10% for all but cytidine, NAR, inosine, NAMN, ATP, NAAD, NADH, and NADP. System RSDs were below 10% for all but cytidine and NAR. Limits of quantification (LOQ) were measured empirically and defined as the concentration producing a signal-to-noise ratio of 10. LOQ were below 100 fmol for all but NMN (1 pmol), ATP (1 pmol), NA (2.5 pmol), and uridine (3.1 pmol). Moreover, the LOQ using this method was at least 3-fold lower for seven metabolites than the previously used method. The previous method is 3-fold more sensitive for one metabolite (Table 3) [3].

**Table 3:** LCMS/MS SRM parameters, sensitivity, and robustness for each metabolite.

Metabolite	Transition (m/z)	CE^a^	Cone volt.	RT (min)	*k’*	LOQ this study (pmol)^b^	LOQ Evans (pmol)^c^	R^2^	RSD^d^ Method	RSD^d^ System
Nam	123 > 96	16	32	9.82	6	0.6	0.45	0.996	2	1
NA	124 > 53	26	32	8.35	5	2.5	1.2	0.999	2	1
Cytidine	244 > 112	18	18	11.14	7	0.01	0.1	0.995	22	16
Uridine	245 > 113	16	18	11.5	7	3.1	1.2	0.99	7	3
NR	255 > 123	12	14	8.98	5.4	0.01	0.2	0.998	4	2
NAR	256 > 124	13	14	10.33	6.4	0.1	0.06	0.948	11	12
Inosine	269 > 137	12	12	12.88	8.2	0.03	0.07	0.974	10	5
CMP	324 > 112	22	16	2.97	1.1	0.09	0.68	1	9	1
UMP	325 > 97	12	20	4.11	1.9	0.06	0.21	0.99	6	4
NMN	335 > 123	12	16	8.92	5.4	1	0.5	1	8	3
NAMN	336 > 124	12	18	4.2	2	0.06	0.18	0.999	10	2
IMP	349 > 137	22	14	9.65	5.9	0.1	0.13	0.995	6	3
ADP	428 > 136	26	30	10.43	6.5	0.03	NIR^f^	0.999	8	3
ATP	508 > 410	16	30	10.51	6.5	1	NIR^f^	0.995	11	3
ADPr	560 > 348	16	26	11.08	6.9	0.02	NIR^f^	0.991	3	4
NAD^+^	664 > 428	26	26	13.64	8.7	0.19	0.17	0.999	8	2
NAAD	665 > 428	24	24	11.89	7.5	0.02	0.26	0.998	12	2
NADH	666 > 649	20	26	12.98	12	0.19	0.06	0.988	10	3
NADP	744 > 604	18	26	12.01	7.6	0.06	0.87	0.996	13	3
NADPH	745 > 729	48	28							

a collision energy

b LOQ of method described in this paper

c LOQ of method in [3]

d RSD expressed as percentage of the mean

### Metabolite Measurement Challenges

In our hands, AMP and NADPH cannot be reliably quantified by these methods. ATP and ADP fragment on source to AMP, similar to NR fragmentation to Nam (Figure 2B Nam extracted ion current). Given poor resolution of AMP from both metabolites, detected AMP signal would represent biological AMP as well as that derived from ATP and ADP. Further, the NADP peak may represent the sum of cellular NADP and a portion of cellular NADPH, which has become oxidized. Thus, care should be taken in interpreting the NADP peak.

Nam is strikingly membrane permeable. Nam in cells can be easily lost into post-cellular supernatants and we suspect that Nam in organelles can be easily lost into post-organelle supernatants.

### Results in mammalian cell line

To test our method on a real sample, we analyzed a glioma cell line, LN428/MPG (a gift of Dr. Robert Sobol, University of Pittsburgh), which had been grown in MEMalpha (10% FBS HI, gentamycin, geneticin) media in triplicate 150 mm dishes (2 x 10^7^ cells per dish by CASY cell count). The results of the analysis are reported in Table 4. As expected, nucleotides, such as ATP, ADP, UMP, and NAD^+^, are high in abundance compared to NAD^+^ precursors and biosynthetic intermediates. The NAD^+^:NADH ratio is ∼39 and the NAD^+^:NADP ratio is 4.6. As expected for cells grown in a type of Dulbecco's modified Eagle's media, Nam but not other vitamins is detectable. The concentration of NMN is low when compared with yeast, suggesting the NMN pool is converted rapidly to NAD^+^ [3].

**Table 4:** NAD^+^/ metabolome of LN428/MPG Cell Line.

Metabolite	LN428/MPG Cells (µM)
ATP	1010 ± 380
ADP	890 ± 150
UMP	370 ± 80
NAD^+^	260 ± 40
Inosine	250 ± 150
Uridine	210 ± 80
CMP	170 ± 70
IMP	98 ± 26
NADP	57 ± 10
Nam	39 ± 2
Cytidine	6.7 ± 4.4
NADH	6.7 ± 2.3
ADPr	6.7 ± 2.2
NMN	1.3 ± 0.3
NA	<4.0
NR	<0.016
NAMN	<0.68
NAAD	<0.24
NAR	<1.1
NAD^+^/NADH	39

## Conclusions

Here, an improved LC-MS method has been developed to quantify the NAD^+^ metabolome. Its principle features are resolution and quantification of 16 metabolites in an alkaline separation, and resolution and separation of 3 metabolites in an acidic separation, both on a porous graphitic carbon stationary phase. The problem of ionization suppression that plagued earlier methods has been eliminated. Preservation and quantification of NADPH remains a challenge.
